# Association Between Residential Segregation and Suicide across the Lifecourse among Black Americans

**DOI:** 10.1093/geroni/igaf122.645

**Published:** 2025-12-31

**Authors:** Eskira Kahsay, Briana Mezuk

**Affiliations:** University of Michigan, Ann Arbor, Michigan, United States; University of Michigan, Ann Arbor, Michigan, United States

## Abstract

Residential segregation is a widely researched structural determinant with negative effects on physical health. However, the neighborhood context also allows for increased social capital and fewer interpersonal experiences of discrimination, potentially benefiting the mental health of those in racially/ethnically homogeneous areas. Still, the impact of segregation on suicide remains unclear, especially among Black Americans whose suicide rates have increased in the last decade. This study investigated the association between segregation and suicide among Black Americans using the National Violent Death Reporting System harmonized to1-year American Community Survey Public Use Microdata Samples (cases N = 10,462; controls N = 1,298,572) in 29 states and D.C. between 2017-2021. In addition to single suicides (N = 6,575), undetermined intent deaths (N = 3,887) were included to account for high rates of suicide misclassification. Black ethnic density (i.e., proportion of Black residents) served as a proxy measure for county-level segregation. Weighted logistic regression models were fit, adjusting for demographics, year, county population density, and county poverty. Stratification by age (early [18-24], young [25-44], middle [45-64], older [65+] adulthood) was conducted. Ethnic density was positively associated with suicide (per 10% increase, OR = 1.13, 95% CI: 1.11-1.14). The association was strongest among middle (OR: 1.31, 95% CI: 1.29 - 1.34) and older adults (OR: 1.18, 95% CI: 1.13 - 1.23). While potentially protective against other dimensions of mental health, racial/ethnic homogeneity may be a salient contributor to suicide risk, particularly later in the life-course, potentially as as a result of accumulated stressors and systemic disadvantages.

